# Characterization of a Cruciferin Deficient Mutant of Arabidopsis and Its Utility for Overexpression of Foreign Proteins in Plants

**DOI:** 10.1371/journal.pone.0064980

**Published:** 2013-05-27

**Authors:** Yimei Lin, Agnieszka Pajak, Frédéric Marsolais, Peter McCourt, C. Daniel Riggs

**Affiliations:** 1 Department of Cell and Systems Biology, University of Toronto, Toronto, Ontario, Canada; 2 Southern Crop Protection and Food Research Centre, Agriculture and Agri-Food Canada, London, Ontario, Canada; 3 Department of Biology, University of Western Ontario, London, Ontario, Canada; Purdue University, United States of America

## Abstract

Plant seeds naturally accumulate storage reserves (proteins, carbohydrates, lipids) that are mobilized during germination to provide energy and raw materials to support early seedling growth. Seeds have been exploited as bioreactors for the production to foreign materials, but stable, high level expression has been elusive, in part due to the intrinsic bias for producing the natural reserves in their typical proportions. To identify mutants governing seed filling, we screened a population of mutagenized Arabidopsis plants for a mutant that failed to fill its seeds. Here we report the identification of *ssp1*, a recessive, viable mutant that accumulates approximately 15% less protein than wildtype seeds. Molecular analyses revealed that *ssp1* is due to the introduction of a premature stop codon in *CRU3*, one of the major cruciferin genes. Unlike many other reserve mutants or transgenic lines in which seed storage protein levels are reduced by antisense/RNAi technologies, *ssp1* exhibits low level compensation by other reserves, and represents a mutant background that might prove useful for high level expression of foreign proteins. To test this hypothesis, we used a bean phytohemagglutinin (PHA) gene as a reporter and compared PHA expression levels in single copy insertion lines in *ssp1* vs. wildtype. These near isogenic lines allow reporter protein levels to be compared without the confounding and sometimes unknown influences of transgene copy number and position effects on gene expression. The *ssp1* lines consistently accumulated more PHA than the backcrossed counterparts, with increases ranging from 12% to 126%. This proof of principle study suggests that similar strategies in crop plants may improve the yield of foreign proteins of agronomic and economic interest.

## Introduction

Historically, bacterial, fungal and animal cell cultures have been exploited to produce chemicals and macromolecules of importance to human health, agriculture, and a variety of industrial processes. The advent of recombinant DNA and transgenic technologies has revolutionized biomolecular engineering to facilitate large-scale production of such molecules. In general, these systems employ bioreactors that necessitate sterility and require considerable capital investment and maintenance expenses. Plants have long been viewed as attractive alternatives to bioreactors, as existing mechanical harvesting, cropping systems, and processing technologies can be employed to reduce infrastructure costs. Additionally, research on the biology of naturally occurring metabolites (e.g. seed storage proteins, lipids, starch) and knowledge of the regulatory control of the expression, modification, maturation, targeting, and catabolism of these macromolecules have informed strategies for plant improvement. Translational research successes have been reported in many areas of agriculture and include yield enhancement [1], food quality [2], pathogen resistance [3], and the targeted expression of specific biomolecules of interest to the fields of medicine, pharmacology and industrial processes. In addition, value-added products such as vitamins [4], vaccines [5], biodegradable plastics [6], and biofuels [7], extend the potential economic and agronomic benefits of transgenic plants.

Numerous approaches for various vector delivery systems, promoter types, and targeting sequences have been explored [8]–[9], and focusing expression of desirable proteins in naturally occurring storage organs such as seeds and tubers has met with some success [10]–[13]. Nevertheless, high-level expression of foreign proteins has proven to be elusive in many cases. Seeds, in particular, may contain processing proteases that may cleave the foreign proteins, reducing the stability/activity of the intended product. The lack of robust expression of transgenes also is due to the apparent bias of the seed towards filling with endogenous reserves. Several lines of evidence suggest a seed filling sensor exists to monitor reserve deposition and composition. For example, transgenic rice harboring a sulfur rich sunflower protein gene express the transgene at relatively high levels, but the total protein and amino acid composition is relatively unchanged due to the downregulation of endogenous reserve proteins [14]. Similarly, Scossa and coworkers [15], found that overexpression of a glutenin gene in wheat resulted in a compensatory reduction in the endogenous prolamins. These and other such studies have revealed that grain quality can be manipulated either by induced mutations or by the expression of specific transgenes. For example, high lysine corn varieties have been developed by exploiting mutations (e.g. opaque 2) that reduce the level of lysine-poor zein proteins, which may also be reduced by employing RNAi technology [16]. Reduction in α zein levels is often associated with rebalancing of the amino acid content to increase the relative proportions of essential amino acids [17]. Likewise, in *Phaseolus vulgaris*, removal of the 7S globulin phaseolin and the lectin phytohemagglutinin results in the up-regulation of several sulphur-rich proteins, and increased levels of cysteine and methionine, at the expense of the non-protein amino acid, S-methylcysteine [18]. In the vast majority of studies, reserve compensation occurs in one of the following forms: enhanced expression of the target protein results in reduced levels of other proteins to maintain either a constant protein level and/or a rebalanced proteome that exhibits a similar amino acid composition profile as wildtype. Alternatively or in addition, manipulation of the levels of one type of reserve material (e.g. lipids) can influence the level of other reserve macromolecules (e.g. starch). These experiments reveal that metabolite/volume sensor(s) exist to monitor and adjust seed reserve contents. Despite evidence for a filling sensor(s), no such molecule has been identified by mutant screens or genomics approaches. An alternative strategy has been employed to reduce the levels of endogenous reserves by employing antisense/RNAi technologies directed against one or more seed storage protein genes/classes. This approach, often coupled with cotransformation of transgenes that express a desired protein driven by a seed specific promoter, has met with success in several different systems. For example, in rice, suppression of both the prolamins and glutelins by RNAi was associated with relatively high level expression of a human growth hormone polypeptide [19]. Similarly, in soybeans, suppression of conglycinin expression was correlated with enhanced expression of a GFP reporter driven by a glycinin promoter [20], yet this same effect was not observed in plants in which both glycinin and conglycinin levels were reduced [21]. In many such studies, detailed analyses of seed reserves are not reported such as to evaluate compensation, and/or the copy number and position effect of transgenes is not presented to unequivocally show that confusing the filling sensor can be exploited to enhance the production of a desirable product.

We screened mutagenized Arabidopsis lines for seeds that are defective in seed filling in an attempt to discover a sensor. To this end, we have identified a viable mutant, *ssp1*, which exhibits low-level compensation, and we describe the testing and validation of *ssp1* for enhancing the expression of a transgene, based on comparisons between single locus reporter isogenic lines in which the expression of the reporter is evaluated in both wildtype and *ssp1* genetic backgrounds.

## Results

### Screen for mutants defective in storage protein accumulation

**Figure 1:**
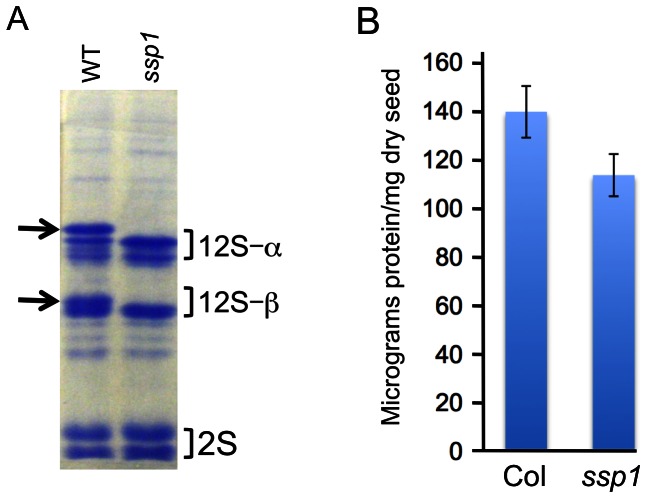
*ssp1* gel profile and seed protein content. A. Mature seeds from Columbia (WT) and *ssp1* were extracted in Laemmli buffer and subjected to SDS-PAGE and staining with Coomassie Brilliant Blue. The 12S cruciferins are post-translationally cleaved into stable a (30–35 kD) and b (21–25 kD) polypeptides, and are predominant components of the gel profile, as are the smaller 2S albumins (2S). The arrows point to two cruciferin polypeptides that are missing from the *ssp1* profile. B. Relative protein levels in mature seed extracts from WT and *ssp1*. Lowry assays were performed on triplicate aliquots of seed extracts and the optical density values were mathematically transformed to yield micrograms of protein per milligram of dry seed, based on the use of BSA as a protein standard. The data represent the average of values from three independent experiments. T-test statistics for *ssp1* vs. wildtype reveal a p-value of less than 0.001.

To identify mutants defective in seed filling and which might represent lesions in sensor/regulatory genes that could be exploited for overexpression of foreign genes, we generated a population of Arabidopsis EMS-mutagenized lines and screened seed protein extracts by gel electrophoresis. The polypeptide pattern of Laemmli buffer extracted seeds is simple due to the abundance of two classes of seed storage proteins, the 2S albumins and the 12S cruciferins. Cruciferins are the major type of seed protein in Arabidopsis, and are encoded by three genes, *CRU1, CRU2* and *CRU3* [22]. The cruciferins are 12S globulins that are synthesized as preproproteins, and are ultimately cleaved into α (30–35 kD) and β (21–25 kD) polypeptides and assembled into hexamers [23]. CRU1 and CRU2 exhibit 70% identity and are of similar size, while CRU3 has an extended glutamine rich region in the center of a larger polypeptide that contributes to its divergence from the other family members (CRU3 exhibits approximately 50% identity to CRU1/CRU2). In general, the posttranslational cleavage events that generate the α and β polypeptides give rise to two distinct clusters of bands when visualized by Coomassie staining of SDS gels. Of over 3000 seed extracts examined, one exhibited a dramatic deviation from the characteristic polypeptide pattern of wildtype seeds (Figure 1). This mutant was designated *ssp1* (*seed storage protein mutant 1*). In the *ssp1* mutant, the largest α and βpolypeptides are apparently missing or reduced in size, while the pattern of the other polypeptides is generally unchanged.

To be potentially useful as a vehicle for overproduction of foreign proteins, *ssp1* or other mutants in which the sensor/filling roles are compromised should fail to fill their seeds properly and as such, would possess increased storage capacity for foreign proteins. To evaluate the potential of *ssp1* as an ‘empty container’, we conducted protein assays on seed extracts from wildtype and *ssp1* seeds, and found that *ssp1* seeds contain approximately 15% less protein than wildtype (Figure 1b). The Students T-test was employed to compare the triplicate values and support the significance of this difference with a p value of less than 0.01.

### Biochemical analyses of *ssp1* and wildtype seeds

**Figure 2:**
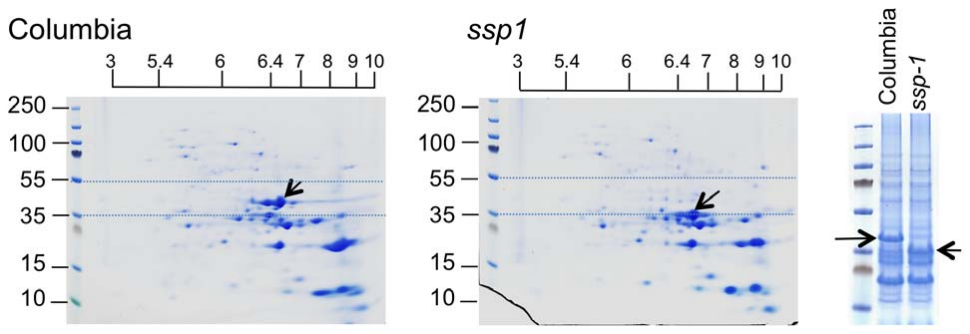
Two dimensional protein profiles of wildtype vs. *ssp1*. Total seed proteins were extracted and equivalent amounts of protein subjected to 2D gel analysis. Note the general concordance of spots in each profile. The major differences (for CRU3, *ssp1* panel; and CRU1, Columbia panel) are highlighted by the arrows.

**Figure 3:**
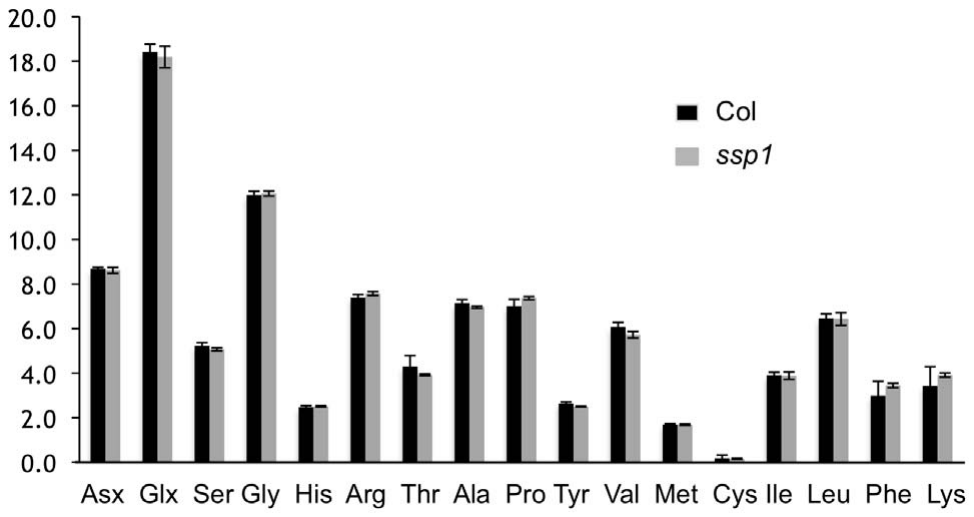
The amino acid composition of *ssp1* seeds is largely unchanged. Total seed protein was extracted from wildtype and *ssp1* in triplicate and analyzed by reverse phase HPLC. The three letter amino acid code applies except to Asx (Asn+Asp) and Glx (Gln+Glu). Statistical analyses revealed no significant changes for any amino acid, suggesting that the loss of the CRU3 component is adjusted by a rebalancing of the proteome.

To more carefully assess the levels of seed reserves in *ssp1*, protein, lipid, amino acid, and starch analyses were undertaken. Quantitative two dimensional gel analysis of phenol extracted seeds and comparison of the staining patterns to the 2-D gel map of Higashi et al. [24], confirmed that the CRU3 bands are missing from *ssp1*, and mass spectroscopy of excised trypsin digested proteins revealed that there is some compensation in *ssp1* by elevated levels of CRU1 (Figure 2 and data not shown). In wildtype plants, CRU3 consists of two prominent bands, though molecular and biochemical analyses (2-D gels followed by immunoblotting) revealed that a variety of processed/phosphorylated forms of CRU3 contribute to polypeptide diversity [25]. In general, the wildtype and *ssp-1* profiles are remarkably similar, with minor differences in protein abundance as gauged by stained gels. Amino acid analyses of total cellular proteins in the two backgrounds was undertaken to determine if the amino acid composition differs (Figure 3). Although it is clear that CRU1 is more abundant in *ssp1* than wildtype (Figure 2), there appear to be only minor changes to the 2D protein profile that lead to a rebalancing of the amino acid composition to be reflective of the wildtype profile. There are minor differences in the levels of many amino acids, but statistically, these differences are not significant. Nevertheless, despite this rebalancing, the level of total seed protein in *ssp1* is significantly lower than wildtype.

**Figure 4:**
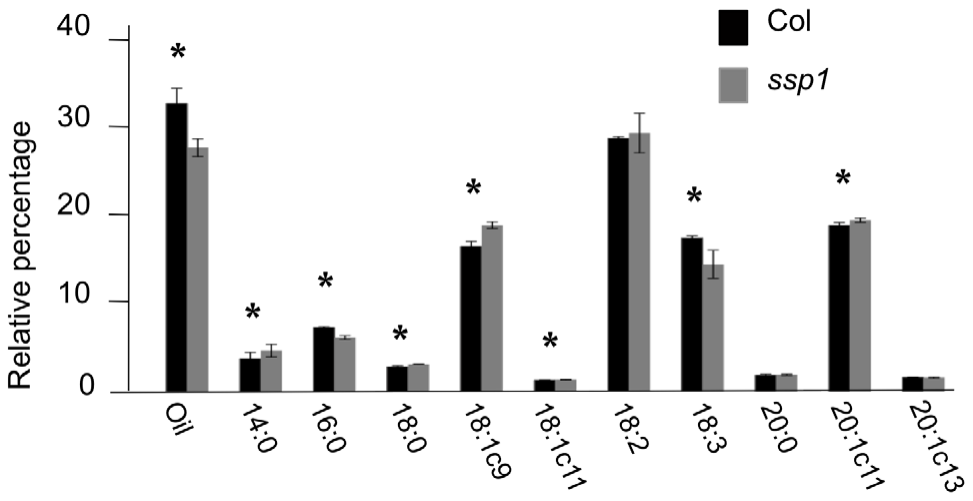
Lipid profiles of wildtype and *ssp1* seeds. Aliquots of wildtype and *ssp1* were analyzed in triplicate by NMR for oil content, and by gas chromatography for the fatty acid species indicated. The oil content of *ssp1* is lower than wildtype, but several fatty acid species are slightly enriched. The asterisks indicate pairwise measurements where T-test statistics revealed significant (p<0.05) differences.

Lipid profiling was conducted to ascertain if the *ssp1* mutation alters oil content or composition. Triplicate samples of both Columbia and *ssp1* were analyzed by NMR for oil content and by gas chromatography for fatty acid composition (Figure 4). Interestingly, *ssp1* contains less oil than wildtype (26.6% vs. 31.5%), yet the percentage of most major classes of fatty acids are slightly elevated in *ssp1* relative to wildtype. Two exceptions are palmitic acid (16∶0) and linolenic acid (18∶3), which exhibit slightly higher levels in wildtype. With respect to the linolenic acid levels, a reasonable conclusion is that the delta 15 desaturase level/activity is reduced in *ssp1*. It is unclear how oil content and composition are altered by *ssp1*, but this observation may be related to a filling sensor effect whereby the protein-to-oil ratio is maintained in the *ssp1* background by a concomitant reduction in lipids.

Starch measurements revealed no significant differences between *ssp1* and wildtype (data not shown). In summary, *ssp1* seeds contain about 15% less protein than wildtype, and exhibit proteome rebalancing to maintain a wildtype amino acid composition. Unlike other naturally occurring or induced seed protein mutants (see the Discussion) which compensate by increasing the levels of proteins or other seed reserves, *ssp1* does not adjust the level of protein reserves to compensate for the loss of CRU3, and thus meets the criterion for a genetic background that might be exploited to overexpress foreign proteins.

### Molecular analyses of *ssp1*

**Figure 5:**
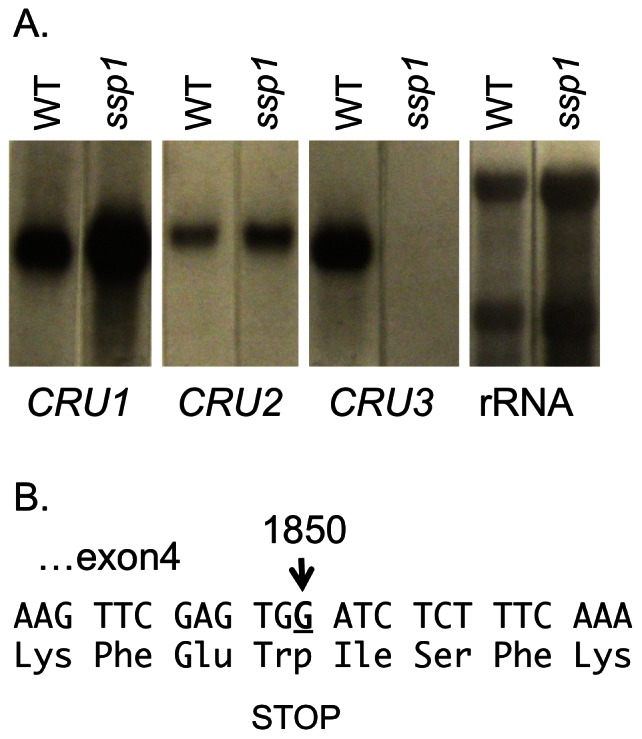
The *ssp1* phenotype is due to a premature stop codon in *CRU3*. Aliquots of total RNA from mature seeds were purified and subjected to RNA gel blot analysis with probes prepared from cloned *CRU1, CRU2, CRU3*, and rRNA genes. *CRU1* and *CRU2* are expressed at approximately equal levels in wildtype and *ssp1*, while there is no *CRU3* signal from *ssp1* mRNA. B. Sequence analysis of the *CRU3* gene in *ssp1* shows that EMS induced a G to A transition mutation in exon 4, converting a tryptophan codon to a premature stop codon.

The absence of one α and one β cruciferin polypeptide in *ssp1* could be due to a lesion in a regulatory gene that governs one of the three *CRU* genes or alternatively, a mutation in one of the structural genes. RNA gel blotting was employed to determine the relative expression of *CRU1, CRU2* and *CRU3* in both wildtype and *ssp1* (Figure 5). *CRU1* and *CRU2* are expressed at similar levels in *ssp1* and wildtype seeds, but the *ssp1* mutant contains no detectable mRNA for *CRU3*. This analysis assigns the largest α andβ polypeptides as products of *CRU3* mRNA translation and indicates that the *ssp1* defect is *CRU3* specific.

In parallel, we conducted map-based cloning of *ssp1*, and observed enhanced segregation of F2 mutants with the MI422 and MI232 RFLP markers and the nga1139 SSLP marker, located on the distal end of chromosome 4 (data not shown). Thus, marker segregation analysis positions the *ssp1* mutation near *CRU3*, which resides at 14.2 Mb. Direct sequencing of the *CRU3* gene from *ssp1* revealed a G to A transition mutation at position 1850 of the genomic sequence, resulting in the conversion of a TRP codon into a premature stop codon. Thus, it is likely that the molecular basis of the *ssp1* mutant is a defective *CRU3* structural gene, and the lack of detectable mRNA implies that nonsense mediated mRNA destruction operates to rid the cell of compromised *CRU3* mRNAs.

To confirm that *ssp1* represents a defect in *CRU3*, we introduced a wildtype *CRU3* gene into *ssp1* and found that the seed protein profile reverted to the wildtype polypeptide pattern (data not shown).

### Strategy for testing the utility of *ssp1* for overproducing foreign proteins

**Figure 6:**
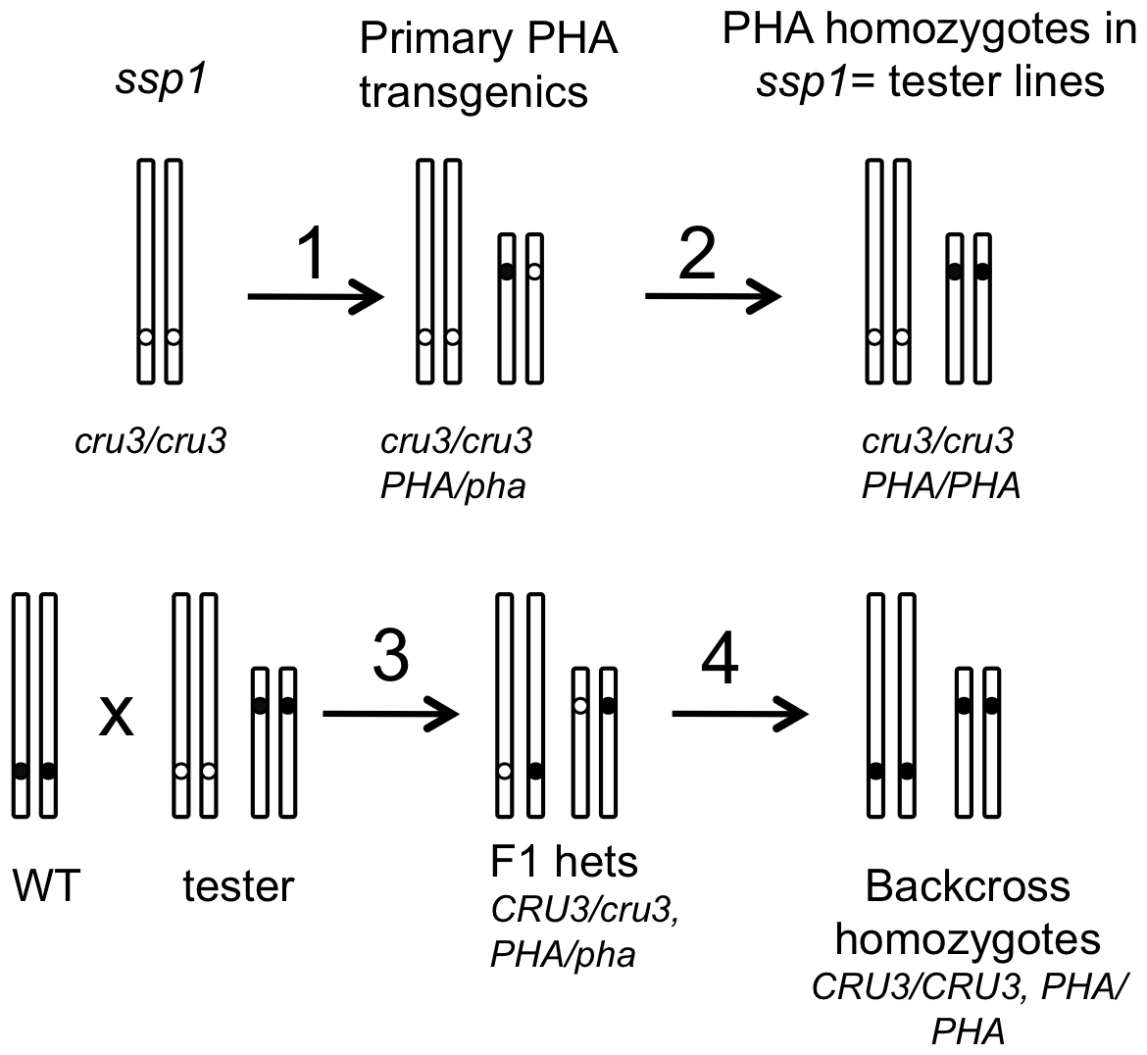
Strategy for testing the empty container hypothesis. Homozygous *ssp1* mutants were transformed with a PHA transgene and single copy insertion lines were selected by Southern blotting (step 1). The primary transgenic lines were selfed and homozygous tester lines were selected by examination of SDS-PAGE profiles of seed extracts (*ssp1* pattern) and 100% hygromycin segregation in the T3 lines (step 2). The tester lines were crossed to wildtype with the resulting F1 plants being both heterozygous for *CRU3 (ssp1)* and for the *PHA* transgene (step 3). Selfing was conducted and F3 lines screened by SDS-PAGE for normal seed protein profiles, by sequencing of *CRU3* to determine homozygosity, and by hygromycin segregation to determine *PHA* homozygosity (step 4). The resulting tester and backcross lines are homozygous for a single PHA insertion such that reporter gene copy number and context are fixed in both the tester (*cru3* background) and the wildtype (*CRU3*) background.

A tenet of our ‘empty container’ hypothesis is that there exists a seed filling sensor mechanism governing reserve accumulation, and that if compromised by a mutation, this genetic background might be exploited to overexpress proteins of economic/agronomic interest. A proof-of-principle strategy was developed to examine the level of a foreign protein in both *ssp1* and wildtype backgrounds in which the transgene copy number and chromosomal context were fixed (Figure 6). The reporter gene employed was the *Phaseolus vulgaris* phytohemagglutinin (PHA) gene, selected for use because *PHA* is a seed specific gene, and the polypeptide is processed and stable within protein bodies of transgenic seeds; in addition, a strong specific antiserum exists that would facilitate expression analyses [26], [27]. Our strategy was to generate primary transgenic lines having a single T-DNA insertion. We employed DNA gel blotting to screen for transgenics that give rise to single hybridizing bands with a PHA probe (see Figures S1, S2, S3). Of 53 independent transformants assayed, 49 possessed multiple insertions, and the four single copy insertion lines were selected and confirmed as single locus sites by observing a 3∶1 ratio of hygromycin resistance∶sensitivity when outcrossed. The primary transformants (*cru3/cru3*; PHA/-; note that with the identification of a premature stop codon in the *CRU3* gene in *ssp1*, the nomenclature now changes to represent *ssp1* as *cru3/cru3*) were permitted to self, and T2 seeds germinated on hygromycin. For transformants bearing a single copy of the PHA gene, the T3 homozygotes would be expected to produce T4 seeds all of which are hygromycin resistant. We identified the T3 lines that were gauged to be homozygous based on hygromycin resistance and used immunoblotting to confirm that these express PHA (data not shown). These lines are genetically *cru3/cru3*; *PHA/PHA*, and were termed the tester lines.

**Figure 7:**
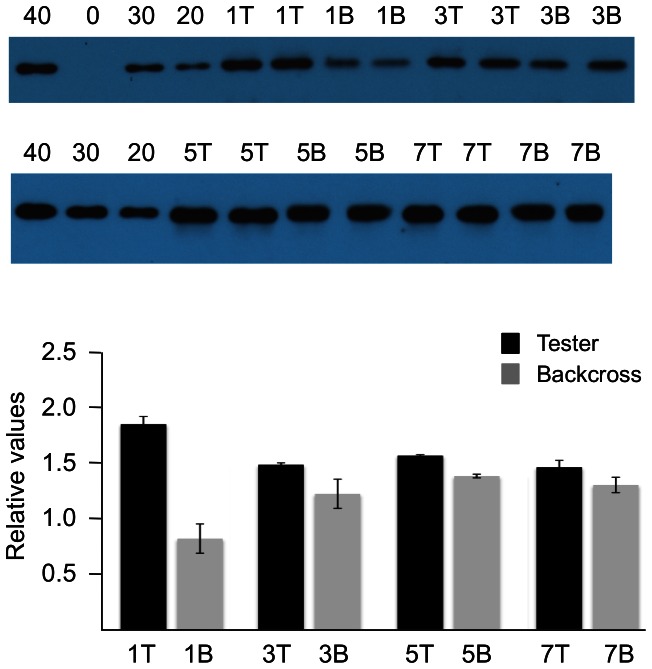
Immunoblotting validates *ssp1* as an empty container. Duplicate one microgram aliquots of total seed protein from tester and backcross homozygotes were subjected to SDS-PAGE and immunoblotting with an anti-PHA antibody. Reporter lines 1, 3, 5, and 7 are shown in duplicate as testers (T) and the corresponding backcrosses (B). Aliquots of purified PHA were included as standards. The autoradiographs were scanned by densitometry and quantified by employing Image J software. The bar graphs represent the average PHA values relative to 30 ng of purified PHA. T-test statistical analyses revealed that lines 1, 3, and 5 have p-values less than 0.05, while the line 7 p-value was greater than 0.05. An independent experiment on the previous generation of these tester/backcross lines revealed similar enrichment trends and p-values.

To unequivocally test the empty container hypothesis, it is necessary to backcross the tester lines with wildtype and to select lines that are homozygous for both *CRU3* and the *PHA* transgene. A three step screening protocol was used to identify such plants. SDS-PAGE was initially used to screen seed extracts for a wildtype protein profile, which would imply either a *CRU3/cru3* or *CRU3/CRU3* genotype (see Figures S4, S5, S6, S7). Homozygotes were distinguished from heterozygotes by direct DNA sequencing of PCR products, such that the single base change in *ssp1* could be discerned. Plants having only a wildtype G signal at the TGG codon were identified and simultaneously examined for hygromycin segregation to determine the zygosity of the PHA transgene. This strategy enabled us to select *CRU3/CRU3; PHA/PHA* plants, which we refer to as the backcross homozygotes. Each reporter line therefore is represented in either an *ssp1* background or a wildtype background in which copy number and position effect variation is fixed by a single PHA reporter transgene. If the *ssp1* mutant could be used to overexpress a foreign (PHA) gene, it is expected that the tester homozygotes would have higher levels of the protein than the backcross homozygotes. Immunoblotting of mature fifth generation seed extracts from four independent lines (1, 3, 5, 7) was undertaken to facilitate this test (Figure 7).

Aliquots of purified PHA were used to construct standard curves and to compare the relative signals in one microgram aliquots of seed protein extracts. This strategy also allowed us to estimate the amount of PHA produced as a percentage of total seed protein. In lines 1 and 3, PHA accounted for 5.54% and 4.44%, respectively, of the total soluble protein, while their backcross derivatives gave levels of 2.45% and 3.66%, respectively. The ratio of these terms indicates that PHA expression in line 1 is 125.9% greater than its backcross counterpart, while line 3 is 21.4% higher. Lines 5 and 7 exhibited less dramatic results with the ratio of differential expression being 13.3% and 12.25%, respectively. Statistical analysis validated lines 1,3, and 5 as significant (p<0.05), while the difference for line 7 was not (p>0.05). Collectively, three of the four lines exhibited enhanced PHA reporter expression in the *ssp1* background, validating the empty container hypothesis.

## Discussion

The use of plants as bioreactors for the economical production of proteins, oils, and specialty chemicals continues to be an area of intense interest (reviewed in [28]). A variety of genetic elements (e.g. promoters, enhancers) have been used to drive the expression of target genes, often in specific organs or tissues [8]. While many successes have been reported, there remain difficulties in obtaining and sustaining high level expression, particularly in storage organs such as seeds. The existence of a seed filling sensor and a natural bias towards the accumulation of endogenous reserves has led to attempts to find mutants that do not fill their seeds, and to antisense/RNAi approaches to genetically reduce the endogenous reserves.

Genetic manipulation of endogenous seed reserves by employing antisense technology has been reported in numerous species, ranging from cereals such as wheat [29], rice [19], and maize [16], [17], to dicotyledonous crops such as soybean [20], [21], and to the model plant Arabidopsis [30]. In some instances, gene expression and proteome changes occur in response to reduced levels of the targeted gene to alter the nutritional quality of the seed [16], [17]. For example, antisense suppression of the C-hordeins of barley resulted in lower levels of these nutritionally poor seed proteins, and the concomitant compensation by enhanced expression of other seed proteins produced seeds with elevated levels of several essential amino acids [31]. In other situations, ‘neutral’ compensation occurs, whereby the enhanced expression of other seed proteins facilitate proteome rebalancing to normalize the amino acid composition of the seed [21], [32].

In antisense lines, the fidelity of the RNAi machinery may be variable in different lines [33], and/or over generations [34], whereas stable mutants with defined roles are expected to yield more consistent results. In addition, some antisense strategies have had unanticipated consequences. For example, targeting of the soybean glycinin mRNAs had an ancillary effect of also reducing the level of conglycinins [21], despite very limited sequence identity amongst the two classes of soybean mRNAs. Nevertheless, this SP- line has been stably maintained for many generations, but the proteome rebalancing that occurs in this line is restricted to endogenous genes and an introgressed glycinin-GFP mimic construct was expressed at levels comparable to the parent lines [21]. This contrasts with a previous report in which the suppression of glycinin created a background in which the glycinin-GFP mimic could be overexpressed [20]. From all of these studies, it is clear that there are differences in the capacity to overexpress transgenes that are dependent on the species, the targets of antisense suppression and the degree to which it manifests its effect, and the localization and amino acid composition of the target protein. Therefore, success in any system will depend upon application of several factors to enhance the expression and stability of the desired trait.

Unlike many genetic backgrounds in which seed storage protein accumulation has been modified, either as a result of a mutation or due to RNAi strategies, *ssp1* is a non-compensating mutant, in the sense that reduced *CRU3* expression/protein levels are not normalized by enhanced expression of other proteins. Although it is unclear why *ssp1* has 15% lower protein levels than wildtype, it is possible that the implied nonsense mediated mRNA decay of mutant *CRU3* transcripts in some way bypasses the filling sensor that activates compensation, resulting in a seed that does not completely fill.

While other reports of reserve mutants or antisense suppression of endogenous reserves have been coupled with analysis of the expression of a transgene [19], [20], [29], [35], our strategy of selecting single copy lines and examining expression of the reporter in near isogenic lines in which the copy number and position of the transgene are fixed, has, to our knowledge, not been reported. In most cases, the transgene copy number has not been investigated, and hence neither its correlation with transgene expression nor the effect of the integration sites on transgene expression can be known. In cases where transgene copy number has been determined, there are many instances in which copy number cannot be correlated with expression levels [27], [30], [36], suggesting that the chromosomal context of the insertion site is a primary determinant of the expressivity of a transgene. In this regard, it has been established that position effect variation may partly be compensated for by employing a matrix attachment region in the transforming construct [37].

Analysis of the four tester lines in our studies clearly show variability in PHA expression, and in three of four lines, reporter expression was significantly higher in the *ssp1* background than the corresponding backcross line. Increases in yield of 10% or greater are agriculturally significant. Our proof-of-principle studies with PHA indicate that it is possible to generate a genetic background that can be useful for overexpression of foreign proteins. It is conceivable that this strategy could be applied to agronomically important members of the *Brassicaceae*, for example, canola. Based on the available data, the targeted reduction of specific seed proteins is likely the most promising strategy and appropriate backgrounds could be identified by screening TILLING lines [38] for specific mutants. Coupling an empty container background that overexpresses a protein of interest with other transgenic approaches, such as the overexpression of the BBX32 gene, proven to increase yield [39], could be a powerful combination in future strategies to combat global food security problems.

## Materials and Methods

### Plant materials and growth conditions

Wildtype and *ssp1* seeds were grown either in a greenhouse under ambient conditions, or in growth chambers at 22°C with a 16 hr/8 hr day/night cycle under fluorescent lighting (100 mE/m^2^)

### Mutagenesis and screening for seed storage protein mutants

Approximately 1 µg of Columbia seeds were suspended in 50 ml of 50 mM phosphate buffer, pH 7.2, containing 0.2% ethyl methanesulfonate and left at room temperature for 16 hours. Seeds were then washed extensively with water, planted in 8 inch pots, and allowed to grow and self in a greenhouse. The M2 seeds were collected en masse and planted at low density in 4 inch pots. Seeds from individual plants were then collected and cataloged, and approximately 100 seeds were ground in hot (>90°C) denaturation buffer (2% SDS, 1% β-mercaptoethanol, 20 mM Tris pH 8.6, 10% glycerol). Following a 5 minute incubation at 95°C, the extract was clarified by centrifugation and the supernatant stored at −20°C until needed. Protein extracts were subjected to electrophoresis on 12% SDS-PAGE gels by standard techniques and protein profiles revealed by staining with Coomassie Brilliant Blue.

### Biochemical techniques

#### Seed protein content

To evaluate seed protein content, 10 mg aliquots of mature seeds were homogenized in 200 µl of hot extraction buffer (1% SDS, 0.5% β-mercaptoethanol, 20 mMTris, pH 8.6, 10% glycerol) and kept at 95°C for 10 minutes, followed by clarification in a microfuge for 5 minutes. One hundred microliters of the supernatant was collected and diluted with three volumes of 20 mM Tris, pH 8.2. Triplicate 25 µl aliquots of the diluted extracts were assayed for protein content by employing the Pierce BCA kit as described by the manufacturer. The raw absorbance values for three independent experiments (each measuring triplicates of both wildtype and *ssp1*) were converted to micrograms of protein/milligram of seed extract using absorbance values of known amounts of bovine serum albumin as protein standards. The converted values were statistically evaluated by using Microsoft Excel, and TTEST p-values were determined.

#### Isolation of Arabidopsis protein for two-dimensional electrophoresis

Total protein from *Arabidopsis* seed was isolated by a procedure from Mooney and Thelen [40] with the following modifications. Dry seed (150 mg) was pulverized to a fine powder in a mortar and pestle using liquid nitrogen. Powder was resuspended in the mortar with 8 mL of homogenization buffer (50% phenol, 0.45 M sucrose, 5 mM EDTA, 0.2% (v/v) β-mercaptoethanol, 50 mM Tris–HCl pH 8.8) with continued homogenization until the homogenate reached room temperature. The homogenate was transferred to a phenol-resistant screw cap tube, mixed for 30 min at 4°C, then centrifuged at 5000 g for 15 min at 4°C. The top phenol layer was transferred to a fresh tube, mixed with five volumes of ice cold 0.1 M ammonium acetate in 100% methanol and placed at −20°C for 2 hrs. Precipitated protein was collected by centrifugation at 5000 g for 10 min at 4°C. The precipitate was washed twice with 20 mL of 0.1 M ammonium acetate in 100% methanol followed by two washes with ice-cold 80% acetone. The sample was then resuspended in ice-cold 70% ethanol, divided into 3 tubes, centrifuged and either stored as a precipitate at −20°C or dried and resuspended for immediate isoelectric focusing.

#### Isoelectric focusing using immobilized pH gradient strips

Protein pellet (from 50 mg of dry seed material) was resuspended in 200 µL of isoelectric focusing buffer (8 M urea/2 M thiourea (deionized), 2% 3-[(3-cholamidopropyl) dimethylammonio]-1-propanesulfonate (CHAPS), 2% Triton X-100, 50 mM DTT, 2 mM Tris (2-carboxyethyl)phosphine hydrochloride (TCEP), 0.8% carrier ampholytes) and incubated for 1 h at room temperature. The sample was centrifuged for 20 min at 14,000 g and the supernatant was transferred into a separate tube. Protein concentration was determined by Bradford assay using BioRad dye reagent (Bio-Rad, Hercules, CA). Protein quantitation was performed in triplicate, and quantitated against a standard curve of bovine serum albumin standard. Exactly 68 µg of protein was added to a separate tube and volume was brought up to 155 µL with IEF buffer/0.001% Bromophenol blue and mixed before pipetting into a loading well of a ZOOM IPGRunner cassette (Life Technologies Inc., Carlsbad, CA). Immobilized ZOOM pH 3–10 NL gradient 7 cm strips (Life Technologies Inc.) were carefully placed into enclosed channel and rehydrated for 18 hrs at room temperature. IPGRunner cell was assembled and isoelectric focusing was performed as follows: 200 V/20 min, 450 V/15 min, 750 V/15 min, 2,000 V/55 min.

#### SDS–PAGE for 2-D electrophoresis

Following isoelectric focusing, IPG strips were incubated in 5 mL 1× NuPAGE LDS sample buffer (Life Technologies Inc.) supplemented with 1% (w/v) DTT for 20 min with gentle agitation, followed by incubation in buffer supplemented with 2.5% (w/v) iodoacetamide for 20 min with gentle agitation. IPG strips were then rinsed with NuPAGE MES SDS running buffer (Life Technologies Inc.) and placed onto 4–12% Bis-Tris ZOOM gels (Life Technologies Inc.). Strips were then overlayed with agarose solution (1× MES SDS running buffer, 0.5% (w/v) agarose). Second dimension SDS–PAGE was conducted in XCell SureLock unit (Life Technologies Inc.) for 43 min at 200 V. Following SDS–PAGE gels were washed with deionized water three times 15 min each and stained with SimplyBlue stain (Life Technologies Inc.). Each *Arabidopsis* seed preparation was resolved by 2-D electrophoresis in at least three independent experiments.

#### Amino acid composition

For amino acid composition analysis, total seed proteins were extracted as described above and precipitated with four volumes of cold acetone. Following centrifugation for 10 minutes, the pellets were dried, and hydrolyzed by the addition of 100 µl of 6N HCl. After 24 hours at 110°C, the acid was removed by vacuum and the hydrolyzates derivatized using PITC. The derivatized amino acids were redissolved in 100 ul of phosphate buffer and analyzed by reverse phase HPLC using a Waters Pico-Tag system at the Advanced Protein Technology Centre, Hospital for Sick Children, Toronto. Triplicate samples of wildtype and *ssp1* were quantified and statistical analyses were carried out using the TTEST function of Microsoft Excel, which revealed no significant differences (p>0.5).

#### Lipid analysis

Triplicate aliquots of mature seeds of both *ssp1* and wildtype were analyzed for oil content by NMR (MARAN Ultra, Oxford Instruments) as described by O'Neill et al. [41] Gas chromatography analysis of seed fatty acid composition was performed as described previously [42]. The triplicate values were statistically evaluated by employing the TTEST function of Microsoft Excel.

#### Molecular techniques

Genomic DNA was purified by a miniprep procedure [43], or by using Sigma GeneElute columns as described by the manufacturer. Total RNA was prepared by employing a Qiagen RNeasy kit. Five microgram aliquots of DNA or RNA were used for DNA/RNA gel blotting. Probes were prepared from DNA inserts of the plasmids harboring the *CRA, CRB, CRC*, and 18S rRNA genes using α ^32^P dCTP (Amersham) and the Rediprime random primer labeling kit (GE Healthcare).

#### Empty container hypothesis testing strategy

*ssp1* homozygotes were transformed with a reporter construct consisting of a 2.8 kbp fragment harboring the *Phaseolus vulgaris* phytohemagglutinin (PHA) gene (pDR214, [27]), which was mobilized into the binary vector pGVPT-HPT [44], and used to transform plants via the method of Martinez-Trujillo et al. [45]. Transgenic plants were selected on 0.5× MS media containing 20 µg/ml hygromycin B (Invitrogen). DNA gel blotting was employed to determine gene copy number by employing a restriction enzyme that cuts at the end of the cloned sequence and therefore single hybridizing bands are indicative of single insertion events (see Figures S1, S2, S3). Single copy lines were selfed and hygromycin segregation examined in both self crosses (100% hygromycin resistant) and backcrosses (75% hygromycin resistance) to confirm the homozygosity of the reporter constructs. These lines are referred to as the tester lines.

The tester lines were backcrossed to wildtype and taken through 3 generations to ensure that both the reporter gene and the wildtype *CRU3* gene were in a homozygous state. The *CRU3* gene region that encompasses the region of the *ssp1* mutation was amplified from these plants by employing the primers: FOR; 5′ATGAGGTCCCACGAGAACATTG3′ and BACK: 5′CGTTAAAGGTAAAGGGACGTGA3′, and the status of *CRU3* assessed by sequencing with the BACK primer (The Centre for Applied Genomics, Toronto). These lines are referred to as the backcross homozygotes.

Seed protein extracts from tester homozygotes and backcross homozygotes were prepared as described above and used for immunoblotting by employing standard techniques. Duplicate one microgram aliquots of seed extracts along with 20,30 and 40 ng of purified PHA (Boehringer-Mannheim) were separated by SDS-PAGE and blotted to nitrocellulose. An anti-PHA antibody [46] was employed, followed by a secondary goat anti-rabbit IgG conjugated to horseradish peroxidase. Detection was facilitated by chemiluminescence, utilizing the Pierce ECL substrate. Multiple exposures on BioFlex x-ray films were taken to insure the linearity of the signals. Autoradiographs were scanned by densitometry and Image J (NIH) was employed to quantify pixel density. Values are expressed as relative to the signals for 30 ng of purified PHA.

## Supporting Information

Figure S1 **Schematic representation of the 2.8 kb** ***PHA*** **EcoRI/HindIII fragment together with the HYG selectable marker.**
(TIFF)

Figure S2 **Analysis of T2 transgenic lines showing that most transformants harbor multiple inserts.**
(TIFF)

Figure S3 **PHA lines 1, 3, 5 and 7 are single locus insertions.**
(TIFF)

Figure S4 **Validation of PHA1 backcross lines.**
(TIFF)

Figure S5 **Validation of PHA3 backcross lines.**
(TIFF)

Figure S6 **Validation of PHA5 backcross lines.**
(TIFF)

Figure S7 **Validation of PHA7 backcross lines.**
(TIFF)
