# Supplementary figures and images for: Characterization of a Cruciferin Deficient Mutant of Arabidopsis and Its Utility for Overexpression of Foreign Proteins in Plants
Source: PLoS One. 2013 May 27;8(5):e64980. doi: 10.1371/journal.pone.0064980 (PMC3664629; doi:10.1371/journal.pone.0064980)

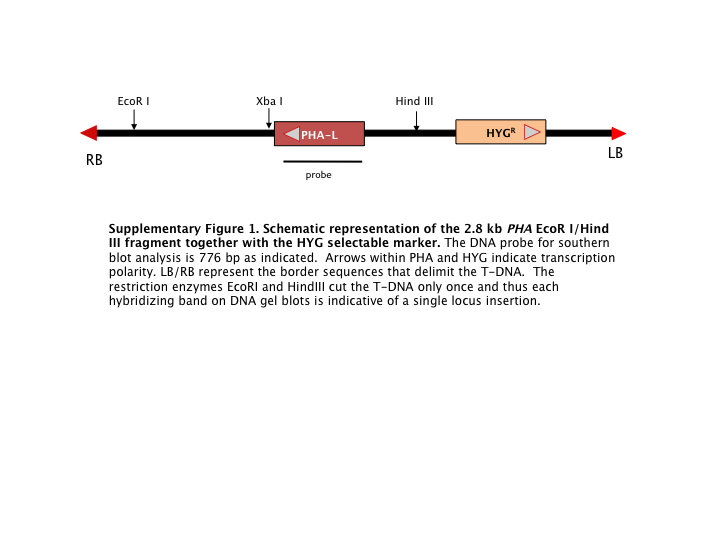

Supplement: Figure S1 — Schematic representation of the 2.8 kb PHA EcoRI/HindIII fragment together with the HYG selectable marker. (TIFF) [file pone.0064980.s001.tif]

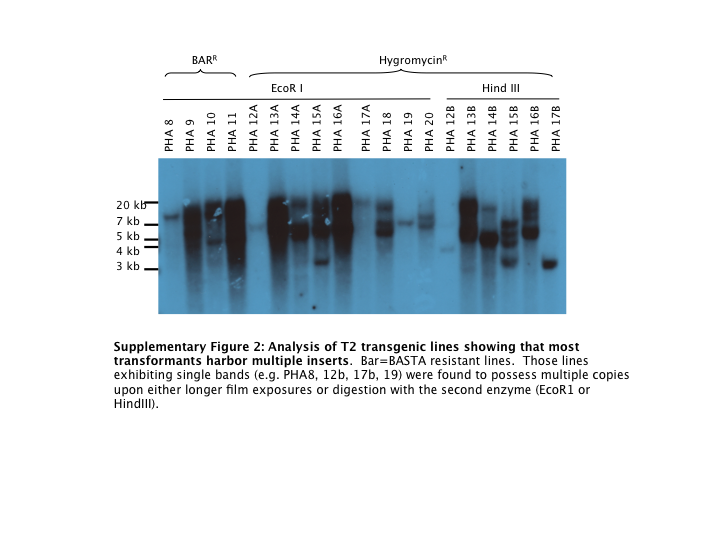

Supplement: Figure S2 — Analysis of T2 transgenic lines showing that most transformants harbor multiple inserts. (TIFF) [file pone.0064980.s002.tif]

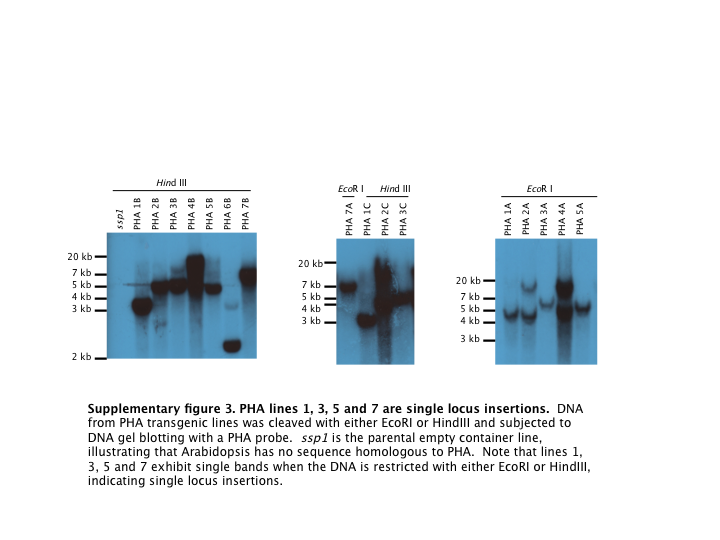

Supplement: Figure S3 — PHA lines 1, 3, 5 and 7 are single locus insertions. (TIFF) [file pone.0064980.s003.tif]

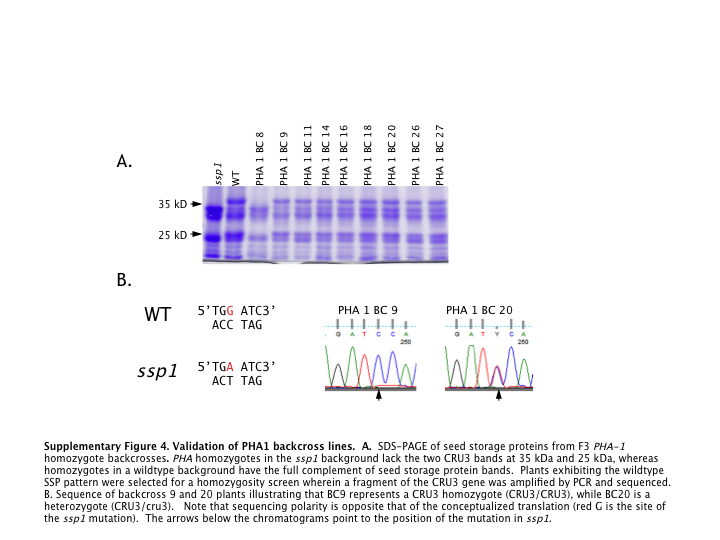

Supplement: Figure S4 — Validation of PHA1 backcross lines. (TIFF) [file pone.0064980.s004.tif]

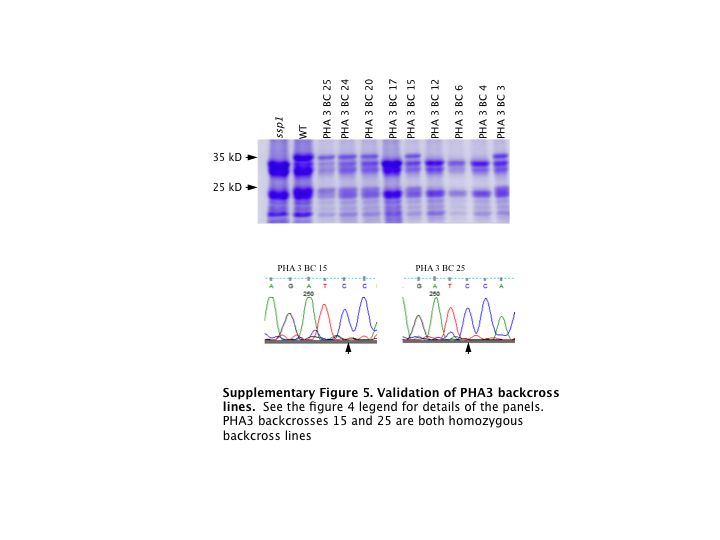

Supplement: Figure S5 — Validation of PHA3 backcross lines. (TIFF) [file pone.0064980.s005.tif]

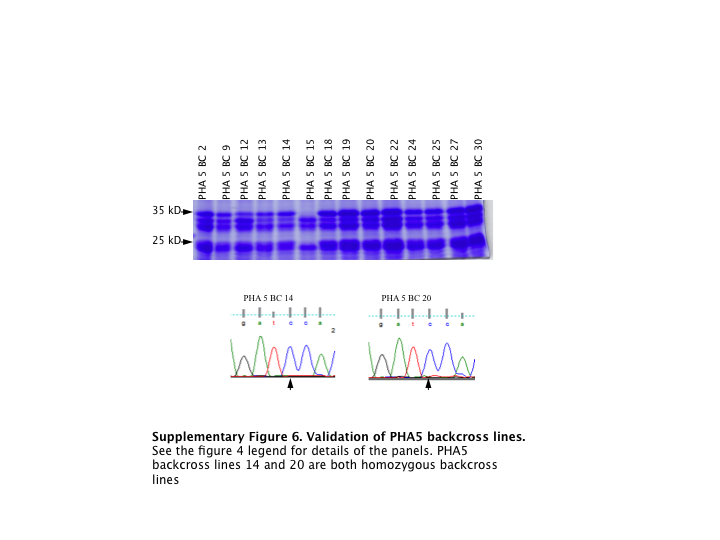

Supplement: Figure S6 — Validation of PHA5 backcross lines. (TIFF) [file pone.0064980.s006.tif]

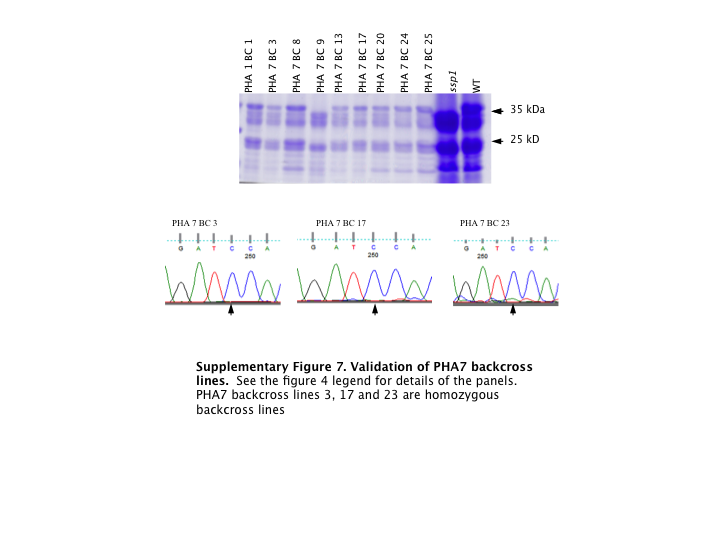

Supplement: Figure S7 — Validation of PHA7 backcross lines. (TIFF) [file pone.0064980.s007.tif]
